# P012. Body image role in medication-overuse headache associated with persistent depressive disorder

**DOI:** 10.1186/1129-2377-16-S1-A111

**Published:** 2015-09-28

**Authors:** Maria Nicolodi, Vanessa Sandoval

**Affiliations:** Foundation Prevention and Therapy Primary Pain and Headache, Florence, Italy

## Background

Body image is a part of each person's self-esteem. Some evidence suggests that body image perception may have direct effects on one's feeling regarding quality of life[1, 2]. Nonetheless, no research has examined the relationship of the impact of body image on medication-overuse headache (MOH) patients suffering also with persistent depressive disorder (PPD). Moreover, the role of body image is stressed in trials concerning mirror therapy[3, 4].

## Aim

We propose that body image improvement can influence both pain perception and PPD in MOH sufferers.

## Materials and methods

The present observational study started in March 2014. Inclusion criteria: 165 women, mean age 43.056±1.3 SD diagnosed as MOH with the ICHD-III criteria and fulfilling DMS-V criteria for PPD. Controls were 106 healthy women, (mean age 43.7±1.59 SD). Women completed affective/cognitive measures of body image (BSQ-track and field). Zung and Hamilton tests were used for scoring depression, MIDAS for perceived quality of life. Each patient was videotaped. Raters, blind to health status, independently rated the attractiveness of the patients. Headache patients with depression reported lower self-esteem, a more negative body image perception than controls; all of them were rated as less attractive by observers when compared to the control group. The multivariate and uninvariate analyses of variance indicated that MOH patients with depression were less satisfied than control subjects. Patients gave their formal consent and underwent aesthetic treatments which included peelings, fillers and polydioxanone stitches.

## Results

Aesthetic medicine improved body image (BSQ - track and field: 0-3 changed from 0 to 2 p > 0.00001), depression (self administered Zung and Hamilton tests decreased, respectively, from 46.2±2.6 SD to 30±2 SD, p > 0.0001; and from 52.6±4.8 SD to 28.4±6.4 SD p > 0.00001), headache pain scores (mean monthly VAS from 8.18±0.5 SD to 4.7±0.7 SD, p>0.00001), and perceived quality of life (MIDAS 0-21+, 15.6±2.4 SD versus 10±0.8 SD p > 0.00001) improved. The improvements were also matched with better rates from the raters (p > 0.01).

## Conclusions

These findings suggest that medical care of body image may induce: A) relief of alteration of body image perceived as a discrepancy between the way sufferers formerly perceived themselves and how they see the changes of their body attributed to excruciating pain and treatments; B) Aesthetic medicine applied, wisely and knowledgeably, using the clinical pharmacology profile of available tools, results in a significant reduction of dissatisfaction, depression and pain perception, as shown by pain score and improvement of perceived condition of severely compromised quality of life.

Written informed consent to publication was obtained from the patient(s).
